# Evaluating search quality and article choice in evidence-based medicine assignments of preclinical students

**DOI:** 10.5195/jmla.2025.2213

**Published:** 2025-10-23

**Authors:** Juliana Magro, Caitlin Plovnick, Joey Nicholson

**Affiliations:** 1 juliana.magro@nyulangone.org, NYU Health Sciences Library, NYU Grossman School of Medicine, NYU Langone Health, New York, NY; 2 caitlin.plovnick@nyulangone.org, NYU Health Sciences Library, NYU Grossman School of Medicine, NYU Langone Health, New York, NY; 3 joey.nicholson@nyulangone.org, NYU Health Sciences Library, NYU Grossman School of Medicine, NYU Langone Health, New York, NY

**Keywords:** Evidence-Based Medicine, Curriculum, Medical Students, Assessment, Librarians

## Abstract

**Background:**

This case report describes the integration of a capstone Evidence-Based Medicine (EBM) assignment into a first-year medical student curriculum and presents an analysis of the correlation between search strategy quality and article selection quality within that assignment.

**Case Presentation:**

A whole-task EBM assignment, requiring students to address a clinical scenario by completing all EBM steps, was implemented after a curriculum-integrated EBM course. Student performance on their search strategy and article selection was assessed using a rubric (1-4 scale). Spearman's rank correlation coefficient was used to assess the relationship between these two variables. Eighty-two students completed the assignment. Fifty-nine percent received a score of 3 for their search strategy, while 77% received a score of 4 for article selection. Spearman's rank correlation coefficient was 0.19 (p-value = 0.086).

**Conclusions:**

While a weak, non-statistically significant correlation was observed between search quality and article selection, the analysis revealed patterns that may inform future instructional design. Educators should consider emphasizing the importance of selecting up-to-date and high-quality evidence and addressing common search errors. Further research, incorporating direct observation and baseline assessments, is needed to draw more definitive conclusions.

## BACKGROUND

Evidence-Based Medicine (EBM) is a critical skill to be developed in medical education training, essential for both school accreditation[1] and performance in USMLE exams[2]. In addition, medical students are required to demonstrate EBM competence prior to residency[3]. Librarians, with their expertise in question formulation, information retrieval, and critical appraisal, are well-positioned to teach EBM. Their involvement in curricula ranges from embedded librarianship in larger courses[4, 5] and leading longitudinal, embedded courses[6–9] to delivering discrete skills workshops[10–12] and offering elective courses[13].

EBM training typically includes instruction on the five steps of EBM: question formulation, evidence retrieval and selection, critical appraisal, application to the scenario, and reflection on performance in the whole EBM task. Each of these steps is necessary for ensuring that medical practice is informed by high-quality evidence [14].

While each step is individually important, researchers suggest that whole-task approaches, in which all the steps are combined in a single task, benefit learning in EBM [15].

In developing instruction and assessment of EBM skills, we sought to understand the relationship between the quality of evidence searches and the quality of the evidence ultimately retrieved, as the ability to effectively search for and select relevant articles is a critical component of EBM. This study examines potential correlations between the quality of students' searches and the quality of their article selections for a whole-task assignment within an EBM course. We hypothesize that search quality (hereafter “search”) and article selection quality (hereafter “selection”) for preclinical medical students are positively correlated.

## CASE PRESENTATION

At NYU Grossman School of Medicine, librarians implemented a capstone assignment after a curriculum-integrated EBM course for preclinical, first-year medical students. Although medical students may have previous experience with research, the EBM course introduces new skills such as how to frame a clinical question, develop effective search strategies, and search biomedical databases efficiently. During the course, they are given instructions and opportunities to conduct searches in different databases.

### Curriculum and Setting

The pre-clinical EBM curriculum at NYU Grossman School of Medicine consists of five required sessions, part of a year-long multidisciplinary course, Foundational Clinical Skills. The sessions are taught about once a month in the second half of the first-year curriculum. In designing the course, we employed different pedagogical strategies proven to help in knowledge retention, including a distributed practice of learning rather than stand-alone courses[16], a whole-task EBM capstone assignment in which students practice all five steps of EBM (Ask, Acquire, Appraise, Apply, Assess) using several clinical scenarios [15], and small-group learning activities.

The first module introduces students to EBM, the second delves into finding evidence, and the remaining modules are devoted to critical appraisal. The three critical appraisal classes are dedicated to the appraisal of cohort studies, randomized controlled trials, and systematic reviews and meta-analyses, all taught in a small group, flipped classroom setting. Before and after this curriculum, students complete a pre- and post-test. After the last critical appraisal class, students complete the whole-task assignment, part of which is the focus of this research. This assignment was embedded in a learning management system, where students submitted their answers and received individual feedback on their performance.

The course lead is a medical librarian and the content director for EBM in the preclinical undergraduate medical education curriculum, having designed and taught these classes for five years. All other facilitators are medical librarians with experience in teaching EBM to a variety of audiences.

### Assignment

As a whole-task activity, this assignment was built so that learners would have the opportunity to practice the constituent components of EBM in an integrated manner. This assignment was mandatory and was set up in the learning management system Brightspace[17] as a quiz. Although students received a grade, this was a formative assessment and did not count towards their final

Foundational Clinical Skills course grade. Learners had about ten days to complete both this assignment and the post-test. After completion, students were given narrative individual feedback.

Since this is taught in the preclinical curriculum, learners practice with clinical scenarios instead of real patients. Based on the scenario provided below, they were free to formulate their own questions and select any article they believed to be appropriate to answer their question. The goal was to select the best evidence to answer the question.

*Your patient is 17 years old and recently has been struggling with suicidal ideation. They are a recreational marijuana user with no history of mental health disorders. You are wondering whether marijuana use might be associated with suicidal thoughts and behavior.*

*Follow the five steps of EBM to examine the best and most current evidence regarding this concern.*

This scenario was used for two reasons. First, the EBM classes in the NYU Grossman School of Medicine curriculum are integrated with the organ systems content. In this case, the assignment aligned with the Brain and Behavior content. Second, the specific topic was based on the number of relevant PubMed articles that address the relationship between suicidal ideation and marijuana use in adolescents. To ensure an efficient evaluation process, we chose a topic that would yield a smaller number of articles when following best practices in database searching. This would prevent assessors from having to review an overwhelming amount of literature, which would significantly delay the feedback response time. During the four years we used this scenario, cohorts of just over 100 students selected only 10 to 15 unique articles. These few articles on the topic have remained relatively consistent from year to year.

To investigate the proposed research question, we focused on analyzing two questions from the assignment:

1. List your final search strategy (keywords AND filters used, if any).
2. Select one article to answer your question.

With these questions, we sought to assess whether there was a correlation between search strategy and the quality of the articles selected.

### Population

The population comprised 105 first-year medical students. From this total, 82 students completed this assignment in spring 2023 and agreed to have their data used for quality improvement reports, such as this one. The data analyzed includes only de-identified datasets from consenting students.

### Answer Scoring

To help reduce bias and improve consistency, we developed a rubric (appendix 1) specifically for this study. This rubric was developed after scoring these assignments for a few years for previous cohorts and applied to the questions described above, and it was revised by the three authors. Additionally, we decided to assess the answers for search and selection separately. By evaluating the two questions independently, we aimed to prevent potential bias from students' responses in the searching questions influencing the assessment of the selection answers.

Only one rater (JM) scored the assignment. Each of the two questions analyzed received a score from 1 (does not meet expectations) to 4 (exceeds expectations). Note that this rubric was developed for this specific scenario and should not be used for other scenarios without adaptations. As mentioned, the literature regarding the relationship between marijuana use and suicidal ideation in adolescents is sparse. In addition, because of the nature of this topic, certain study designs are not feasible. Hence, the available systematic reviews and meta-analyses would constitute stronger evidence than one observational study alone — we want to underscore that this is not the case for all clinical questions.

### Statistical analysis

Because the data consists of ordinal data, we used Spearman's rank correlation coefficient to test whether there was a relationship between the two datasets. The test's goal is to assess whether the change in the magnitude of one variable is associated with a change in another's, either in the same (positive correlation) or the opposite (negative correlation) direction [18].

### Results

**Figure 1:**
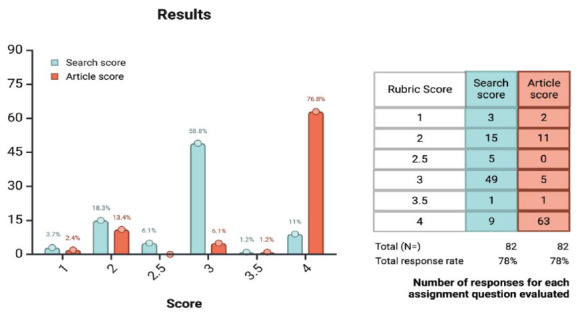
Distribution of Scores for Search Quality and Article Selection (unmatched). Created with BioRender.com.

Eighty-two students completed this assignment. The possible scores for searching were posed as a scale ranging from one (does not meet expectations) to four (exceed expectations). As the rubric shows (appendix 1), there was the possibility of a 0.5 points deduction for pre-established events. Most learners (about 59%) received a score of three, while only 11% received the maximum score of four (figure 1).

The same grading scale (1-4, with possible deductions) was applied to the evidence selection rubric. For this question, most learners (about 77%) received the maximum score of four, exceeding expectations. These students selected a recent systematic review related to the scenario.

Table 1 lists the articles chosen by these students and the number of times that title was selected: 82 students chose 16 different articles. Thirty-two students (39%) selected the two most current systematic reviews on this topic at the time. Interestingly, learners also selected book chapters, cross-sectional studies, and older articles, although less frequently — these types of articles do not correspond to the best, most current evidence.

**Table 1:** Types and Frequency of Articles Selected by Students.

First Author	Title of Article	Type of Article	Year of Publication	Number of Times Selected
Gobbi G [19]	Association of cannabis use in adolescence and risk of depression, anxiety, and suicidality in young adulthood: A systematic review and meta-analysis	Systematic Review and Meta-Analysis	2019	28
Fresán A [20]	Cannabis smoking increases the risk of suicide ideation and suicide attempt in young individuals of 11-21 years: A systematic review and meta-analysis	Systematic Review and Meta-Analysis	2022	6
Flores MW [21]	US trends in the association of suicide ideation/behaviors with marijuana use among adolescents ages 12-17 and differences by gender and race/ethnicity	Cross-sectional study	2023	3
Kahn GD [22]	Marijuana use is associated with suicidal ideation and behavior among us adolescents at rates similar to tobacco and alcohol	Cross-sectional study	2022	2
Zhang X [23]	Suicidal ideation and substance use among adolescents and young adults: a bidirectional relation?	Cohort Study	2014	2
Lydiard JB [24]	Prospective associations between cannabis use and depressive symptoms across adolescence and early adulthood	Cohort Study	2023	1
Athanassiou M [25]	The clouded debate: A systematic review of comparative longitudinal studies examining the impact of recreational cannabis legalization on key public health outcomes	Systematic Review	2023	1
Carvalho JV [26]	Association between cannabis use and suicidal behavior: A systematic review of cohort studies	Systematic Review	2022	1
Basith SA [27]	Substance Use Disorders (SUD) and suicidal behaviors in adolescents: Insights from cross-sectional inpatient study	Cross-Sectional Study	2021	1
Bolanis D [28]	Cannabis use, depression and suicidal ideation in adolescence: Direction of associations in a population based cohort	Cohort Study	2020	1
Sellers CM [29]	Substance use and suicidal ideation among child welfare involved adolescents: A longitudinal examination	Cross-sectional Study	2019	1
Bahorik AL [30]	Medical and non-medical marijuana use in depression: Longitudinal associations with suicidal ideation, everyday functioning, and psychiatry service utilization	Randomized Controlled Trial (secondary data)	2018	1
Gilder DA [31]	Depression symptoms associated with cannabis dependence in an adolescent American Indian community sample	Cross-Sectional Study	2012	1
Prescrire Int [32]	Adverse effects of cannabis	Review	2011	1
Pedersen W [33]	Does cannabis use lead to depression and suicidal behaviours? A population-based longitudinal study	Cohort Study	2008	1
Resnick MD [34]	Protecting adolescents from harm: Findings from the national longitudinal study on adolescent health	Cross-sectional Study	1997	1

To better visualize the scores, we created a contingency table (Table 2). The data shows that 40 students who scored 3 in their search received a score of 4 for their article selection. In addition, 11 students with a search score of 2 achieved the highest score for the article selection. Three students with the lowest search score failed to select an appropriate article to answer their question, receiving scores of 1 or 2 for their article.

**Table 2:** Contingency Table of Search Quality and Article Selection Scores. ⊙ blue: lower search scores and equal or higher selection scores; * green: higher search scores and equal or higher selection scores; - magenta: search scores higher than selection scores.

	Article Selection 1	Article Selection 2	Article Selection 3	Article Selection 3.5	Article Selection 4
Search 1	⊙ 1	⊙ 2			
Search 2		⊙ 2	⊙ 1		⊙ 11
Search 2.5		− 1		⊙ 1	⊙ 4
Search 3		− 6	* 3		* 40
Search 3.5	− 1				
Search 4			− 1		* 8

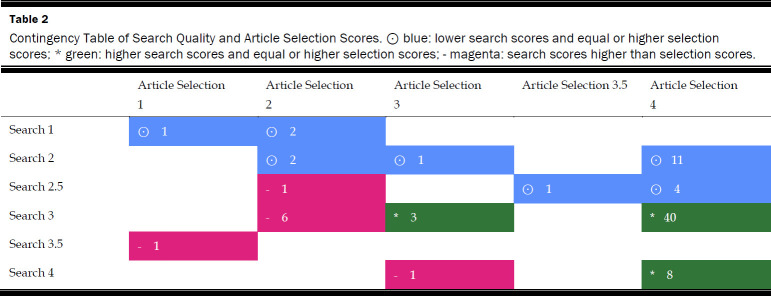

We used three symbols to denote three different scoring patterns: high search scores (≥ 3) with an equal or higher selection score (denoted as *, green), lower searching scores (≤ 2.5), with equal or higher selection scores (denoted as ⊙, blue) and search scores higher than selection scores (denoted as -, pink). Fifty-one students (62%) who scored 3 or more in their search had an equal or higher score in their article selection (*). For students with lower search scores, there was also a trend where their article selection scores were equal or higher than their searches (⊙). Only nine students (11%) had a search score higher than their selection score (-).

The table shows that 62% of high scoring students (green * cells) scored equal or higher in article selection compared to searching, while 26% (blue ⊙ cells) of low scoring students had an equal or higher score in article selection. Among students who scored 3 or higher in their search, 86% (51 students) also scored 3 or higher on article selection. Conversely, 69% (16 students) who scored 2.5 or lower in searching achieved 2.5 or higher in article selection, with an absolute difference of 17%. To assess the relationship between these variables, we calculated the Spearman's correlation coefficient. The result (r=0.19, p-value = 0.086) indicates a weak, positive correlation that is not statistically significant.

## DISCUSSION

This study attempted to determine whether there was a correlation between the quality of searches and the quality of article selection for a whole-task EBM assignment for preclinical medical students. We hypothesized that there would be a trend where higher scores in searches would lead to higher scores in selection. After analysis, the data showed that there was a weak, positive correlation between these two questions, but the findings were not statistically significant (p-value = 0.086).

The contingency table, however, provides some insight into the relationship between search quality and article selection based on this assignment. The fact that a portion of students with lower search scores (≤2.5) still managed to select appropriate articles suggests that students may be compensating for poor search skills through browsing, searching in other databases, or using other strategies. In addition, many students had article selection scores equal to or higher than their search scores. This could indicate that students are better at selecting appropriate articles than they are at searching. It is also possible that the assignment design facilitated good selection even with suboptimal searches, as we selected a topic with narrow available evidence on purpose.

Several practical implications arise from this study. First, instructors should explicitly address the issue of students selecting older articles, book chapters, or cross-sectional studies when more current and less prone to bias evidence is available. Instructors should consider providing specific instruction on how to identify the most up-to-date and high-quality evidence, which should be reinforced throughout the curriculum if possible. This is a change we implemented in our curriculum following this assessment. Second, even though the correlation between search quality and article selection was not statistically significant, the data suggests that search skills may influence article selection. Therefore, instructors should continue to emphasize and reinforce searching skills: this should help both in student learning and in providing more robust data for future studies. Third, educators should be mindful of the scope of the topic chosen for EBM assignments, as this can impact the results. A broader topic with more diverse evidence could provide a more realistic and challenging learning experience. Based on this reflection, we plan to change the assignment topic in future cohorts. Fourth, we encourage educators to systematically assess students' searching patterns. We observed certain behaviors that could impact the database results negatively (e.g., use of quotation marks for all keywords in PubMed, disabling Automatic Term Mapping, improper use of Boolean operators, lack of appropriate synonyms, etc.), which are now addressed in advance. Lastly, we note that both this capstone assignment and the post-test were released and due on the same day through the Learning Management System, causing some confusion among students who thought that there was only one activity to complete. As a result, several students were missing either the assignment or the post-test. Therefore, we recommend that separate tasks should have different release and due dates to avoid confusion.

This study has several strengths. First, it addresses a gap in the literature by focusing on the relationship between search quality and article selection. Researchers have noted that the focus on critical appraisal rather than the other EBM steps is a shortcoming of this literature[14] and this study adds evidence to the assessment of earlier steps. Second, the use of a whole-task assignment, where students were free to formulate their own questions and searches, adds to the validity of the study. Third, a rubric was used to assess search quality and article selection, helping reduce bias and improve consistency. Finally, the study is based on a real-world EBM assignment, part of a medical school curriculum, and highlights that continuous assessment and reflection improves curriculum, as discussed in the paragraph above.

This study also has several limitations that should be considered. We assessed only one cohort of students from a single institution, which may limit the generalizability of our findings. Furthermore, the assignment did not include direct observation, making it challenging to understand the context, idea generation, browsing patterns, and search formulation and reformulation tactics that students might have employed. An additional limitation is that the rubric was not formally validated and was designed based on the topic chosen. This narrow topic may have influenced the range of articles available for student selection. Finally, there could be potential confounding variables that might have influenced the results, such as prior knowledge or experience with database searching, which were not measured as a baseline.

Future research should incorporate direct observation of the search and selection process in order to better capture student performance. Although challenging, conducting longitudinal studies that follow students throughout their medical education could help provide insights into how their search and selection skills develop over time. Such studies could assess the long-term impact of EBM training on students' abilities and identify key points in the curriculum where reinforcement or additional instruction is most needed. Another helpful assessment would be to evaluate students' prior knowledge and experience with database searching before starting EBM classes. With this type of data, researchers could also investigate whether certain groups of students benefit more from specific types of training. Another possible future direction is analyzing the performance of students with high search scores but low selection scores, and vice versa. This could elucidate factors that could contribute to efficient or inefficient evidence selection in the EBM process. Finally, with the increasing use of AI in literature searches, future studies could examine how the use of AI tools can impact students' search and article selection compared to traditional methods.

## Data Availability

The data that support the findings of this study are openly available in Zenodo at https://doi.org/10.5281/zenodo.15683435.
