# Supplementary material for: Evaluating search quality and article choice in evidence-based medicine assignments of preclinical students
Source: J Med Libr Assoc. 2025 Oct 23;113(4):366–73. doi: 10.5195/jmla.2025.2213 (PMC12604067; doi:10.5195/jmla.2025.2213)
Supplement: Supplementary file 1 — Appendix A [file jmla-113-4-366-s01.docx]

Appendix 1: Rubric

|  | 4 (Exceeds expectations) | 3 (Meets expectations) | 2 (Meets some expectations) | 1 (Does not meet expectations) |
| --- | --- | --- | --- | --- |
| Searching* | At least 4 database searching concepts: Boolean operators; Keywords related to the question; synonyms; filters | 3 concepts | 2 concepts or less | Did not search a database, or did not report search, or used natural language question verbatim |
| Article selectionº | Selected a Systematic Review or Meta-analysis (related to the question/scenario) § | Selected a prospective cohort study (related to the question/scenario) | Selected a cross-sectional study, or a retrospective cohort study (related to the question/scenario) | Selected a review article; or article selection was out of scope; or article was not current enough for this topic |

*For database searching **0.5 point was deducted if** student (cumulatively):

- Used [ ], “” or a different symbol instead of parentheses
- Used &, + or a different symbol instead of Boolean operators
- Did not use parentheses, but added synonyms
- Added too many irrelevant keywords. “Too many” may depend on personal judgement, but here are examples of when this is applied:
  - effect"[All Fields] OR "effecting"[All Fields] OR "effective"[All Fields] OR "effectively"[All Fields] OR "effectiveness"[All Fields] OR "effectivenesses"[All Fields] OR "effectives"[All Fields] OR "effectivities"[All Fields] OR "effectivity"[All Fields] OR "effects"[All Fields])

§ For Systematic Reviews **0.5 point was deducted if**student:

- selected a SR where the outcome and population were marginally studied (not the main focus of study), e.g.:
  - Athanassiou M, Dumais A, Zouaoui I, Potvin S. The clouded debate: A systematic review of comparative longitudinal studies examining the impact of recreational cannabis legalization on key public health outcomes. Front Psychiatry. 2023;13:1060656. Published 2023 Jan 11. doi:10.3389/fpsyt.2022.1060656
